# Studying Wind Chill Index as a Climatic Index Effective on the Health of Athletes and Tourists Interested in Winter Sports

**DOI:** 10.5812/asjsm.34861

**Published:** 2010-06

**Authors:** Gholamreza Roshan, Gafar Mirkatouli, Ali Shakoor, Vahid Mohammad-Nejad

**Affiliations:** 1 Department of Geography, Faculty of physical geography, University of Tehran, Tehran, IR Iran; 2 Department of Geography and Urban Planning, University of Golestan, Gorgan, IR Iran; 3 Department of Geography, Islamic Azad University, Marvdasht Branch, Marvdasht IR Iran; 4 Department of Geography, University of Urmia, Urmia, IR Iran

**Keywords:** Climate change, Wind chill index, Winter Sports, Athletes' Health

## Abstract

**Purpose:**

Estimating wind chill index as one of the indexes effective in body comfort, specifically for athletes and tourists interested in winter sports.

**Methods:**

Meteorology data including temperature and the percentage of relative humidity of 6 synoptic stations of Chaharmahal-Bakhtiyrai province, Iran from 1990 to 2007 were extracted from Iranian Meteorology Site. In order to calculate the values of wind chill, the innovative formula of NOAA Meteorology Services Center [T (WC)= 35.74+0.6215T-35.75V+0.4275TV] was used.

**Results:**

After analyzing wind in all stations, it became evident that the great percentage of wind calm related to fall, and spring had the most wind distortions. In studying the mean temperature during this studying period, Koohrang station with mean of 9.8°C was identified as the coldest station and Lordegan with a mean of 17°C represented the warmest station of the region observed. According to degrees derived from wind chill index, Koohrang station in January with a mean of −28.75 was known as the coldest and roughest station.

**Conclusion:**

Among the studied stations, Koohrang had the most intensive degrees of wind chill occurrence and Lordegan had the calmest conditions. Therefore, athletes and tourists should use warmer clothes and covers in cold seasons in Koohrang in comparison with other studied regions, in order to protect themselves from the negative effects of sudden cold and occurrence of intense wind chills.

## INTRODUCTION

The word “Wind Chill” has been coined for the first time in an article by Dr. Saypel in 1939 named as “Adaptation of South Pole discoverers with cold weather“. Later in the process of discovering polarregions, Saypel and his colleague, Pasel, conducted several studies regarding quantifying and estimating the values of wind chill. They presented a parameter based on their findings which is still considerable in the background of this discussion^[1]^.

Feeling temperature by human body changes based on variations in atmospheric conditions. In order for the body temperature to remain stable, the temperature absorbed and produced by the body should be the same as the lost temperature. If the lost temperature is more than the absorbed and produced temperature, the human body feels cold and vice versa. Therefore, there is a constant exchange of temperature between human body, specifically at skin surface, and the surrounding environment. In a cold day, a thin layer of hot weather molecules covers the skin and protects the body against the surrounding cold weather, and decrease the speed of heat transmission to the environment^[2, 3]^.

When wind blows, the thin layer of hot weather molecules get away from body and the body heat is quickly transmitted to the environment, and the body feels cold. The faster the wind blows, the faster the body loses heat and feels cold. The fact that how wind can cause human body feels colder is described through an index called the wind chill-producing. In other words, the common effect of wind and temperature in loss of heat in human body and other organisms is called wind's chill-producing or wind chill^[2]^.

This parameter is presented to express the relationship between the lack of human comfort and the common effect of wind and temperature. The above parameter is not an actual temperature, however; this is the feeling of temperature by body that is measured based on the simultaneous effect of wind and the temperature on the body. This parameter converts the frost-chill-producing power of the wind to temperature equivalence in a clear day (in which the wind speed is equal to or less than 6.4 km/hr^[4]^.

Intense blow of wind at temperatures lower than zero could emit heat from body quickly that might lead to skin frostiness. In a cold weather, skin humidity can also be an important factor in the way cold is felt since water on body skin directs the loss of heat in a better way. The high speed of energy loss by body might lead its temperature to under normal level and cause a condition that is called hypothermia. This condition leads to body tiredness and faintness and if body temperature gets lower than 26° C (degree centigrade) it causes death^[2]^. Temperature alone cannot provide us enough information to decide on the type and number of clothes we wear. Other climatic factors specifically wind have significant role in this regard. The importance of wind chill index (WCI) is that it can be used as an appropriate criterion for wearing clothes in cold weather^[5]^.

Although this index has not taken into account some environmental factors such as relative humidity of weather and sun radiation, the researchers working on and conducting research on this index believe that using this index is sufficient in order to protect human health in cold weather. For example, meteorology organizations of many countries presents the wind chill-producing degree to people through public media and internet sites in order to choose covering and warm clothes. Besides, the US army uses this index for developing and reforming the production of warm uniforms for soldiers^[6]^.

Wind chill index has wide range of applications in countries located in cold regions, in a way that an opinion poll performed in Canada shows that 82% of the people of this country use wind chill index in order to plan their daily outdoor activities in winter. Furthermore, many organizations and schools consider this index while planning and making decisions. Even people who make living outdoors like constructional workers, hockey club workers, or operators of telecabins, stop the work when this index is very low^[7]^.

Table 1 presents the economic loss of the world due to natural disasters from 1900 to 2001. According to this table, wind chill which is very similar to cold wave in its nature, is among the first eight economically destructive events in the world^[8]^.

**Table 1:** The amount of economic destruction of natural disasters from 1900 to 2001^[8]^.

Type of Loss	Price of Loss
**Earthquake**	$248,624,900,000
**Flood**	$206,639,800,000
** Tropical Cyclones**	$80,077,700,000
**Windstorm**	$43,890,000,000
**Fire**	$20,212,800,000
** Drought**	$16,800,000,000
**Cold Wave**	$9,555,000,000
**Heat Wave**	$5,450,000,000
**Sum total**	$631,250,200,000

**Research background related to innovative formulas to estimate WCI:** Different formulas for estimating wind chill have been presented so far that some of them are pointed out here. Variables related to WCI are subjected to some changes in different tables which are due to using different formulas for estimating heat loss or different conceptions of heat loss from body or slow wind speed.

In the 1930's, Sypel and Pazel presented a formula for estimating the heat loss from human body based on their investigation in Southern Pole ^[3, 9]^:

Equation 1:

H=(10.45+10V−V)(33−T)

In the above formula:

**H:** heat loss from un-covered body skin based on kcal (m^2^)/h

**V:** wind speed based on ms

**T:** temperature in °C

They presented the following formula by using the formula 1 for the equivalent temperature (ET) of the lost energy^[9]^.

Formula 2:

WCT=33+(T−33)(0.474+0.454V−0.454V)

In the above formula:

**WCT:** equivalent temperature of the lost energy in C (in other words a temperature of human body in which energy loss is almost H calories)

**V:** wind speed in ms

**T:** temperature in ° C

Formula 2 is true when: T<33 ° C and V>1.79 m(s). From other methods innovated for estimating wind chill, the method presented by Oshezovaki and Bloestin in 2001 could be mentioned. This formula that is innovated using the progress of science and technology and computer models is:

Formula 3:

W=13.12+0.6215T−11.37V+0.3965T×V

In the above formula w is the value of wind chill in ° C, T is temperature in c, and V is wind speed at scale of km/h^[10]^.

The wind chill formulas being used based on the formula of Saypel and Pazel are traditional formulas. In this formula temperature and wind speed are used for estimating heat loss^[7]^.

Formula 4:

H=(10.45+10×V−V)×(32−T)

In this formula, heat loss is based on kilo calorie multiply by sq/m and hour, T is temperature in C and V is wind speed based on meter over second. In order to convert formula 4 to modern units of watt over sq/m, formula 5 is used.

Formula 5:

H(w/m)=12.1452+11.6222×V−1.16222×(33−T)

In the US and Canada, formula 6 is used for estimating heat loss through temperature and wind speed.

Formula 6:

Te=S−(S−T)×(0.474266+(b×V)+(C×V)

In this formula, Te is Equivalent temperature, S is skin temperature (33 °C or 91.4 °F), the b and c values are dependent on units used for estimating wind and they change based on variations of related units according to Table 2. The traditional formula used for estimating temperature of wind chill that was applied by National Oceanic and Atmospheric Administration (NOAA) International Meteorology Center has shown wind chill temperature lower than the formula used today. This traditional formula is illustrated accordingly in Fahrenheit and Celsius through formulas 7 and 8 ^[11]^.

Formula 7:

T(WC)=0.0817(3.71V+5.81−0.25V)(T−91.4)+91.4

Formula 8:

T(WC)+0.045(5.27V+10.45−0.28V)(T−33)+33

**Table 2:** b and c values based on chosen units of wind speed

b	c	Unit
0.453843	−0.0453843	m/s
0.239196	−0.0126067	Km/h
0.325518	−0.0233477	kt
0.303444	−0.0202886	Mi/h

In both formulas 7 and 8, **T (WC)** is wind chill temperature, **T** is temperature, and **V** is wind speed in m/hr.

Recently at NOAA Meteorology Service Center, a new formula has been presented that is more actual for estimating WCI than previous ones. In this formula, two factors of temperature and wind are used ^[11]^. In this formula, temperature is stated at Fahrenheit and wind speed is stated at meter^[12]^.

Formula 9:

T(WC)=35.75+0.6215T−35.75V+0.4275TV

Recently, this modern modified formula is used in all meteorology centers^[13]^.

**Research Purpose:** In spite of the attention paid to and significance given to WCI in other countries, especially in the last two decades and also the necessity of conducting such research felt throughout the country, a comprehensive and extensive study in this area has not been carried out in Iran. Although Iran, like other countries such as Scandinavia and Canada, is among countries located at high latitudes and it might be less affected by the wind chill phenomenon, some specific parts of the country such as snow-clad heights, snow-ski centers that are pleasure ground of athletes and tourists, encounter wind chill more.

Therefore, the purposes of this research are as follows:

1. Estimating WCI as one of the indexes effective in body comfort, specifically for athletes and tourists interested in winter sport and encounter lower temperatures more often.
2. Identifying and studying the probability of occurrence and return period of this phenomenon. In other words, by correct application of occurrence probability and reoccurrence of wind chills for planning and decision making in probable crisis we could make a significant attempt toward controlling the damages caused by this natural disaster and decreasing its effects on athletes and tourists interested in winter sports.

## METHODS AND SUBJECTS

**Fig. 1:**
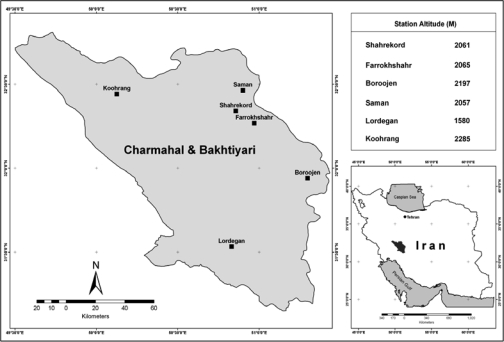
The study area, Chaharmahal & Bakhtiyari Province in Iran

Due to the reason that formulas related to wind chill generally do not have any application in hot regions, the studied stations were chosen from mountainous cold and semi-cold regions of Zagros Mountains in western part of Iran. Besides, based on the aforementioned reason, it is not possible to estimate wind chill in hot seasons (spring and summer). Therefore, for conducting this project synoptic stations of Charmahal & Bakhtiyari province including stations of Shahrekord, Koohrang, Lordegan, boroojen, Saman, and Farokh Shahr were used (Fig. 1). Also according to the common statistical period, winter season of the years 1990 to 2007 were chosen as samples.

For estimating wind chill, the innovative formula of NOAA International Meteorology Service Center, formula 9, was used. Therefore, in the selected stations the monthly average temperatures were analyzed in Fahrenheit and monthly average of wind speed was analyzed in m/hr. Since one of the major purposes of this study is to apply the results in crisis management in future for decreasing the destructive effects of this phenomenon on health of athletes and tourists interested in winter sports, estimating the degrees of wind chill for different statistical periods was followed by several formulas used for estimating the probability of occurrence and reoccurrence of wind chills with different intense degrees.

## RESULTS

After analyzing wind in all stations it became evident that the great percentage of wind calm relates to fall, and spring has the most wind distortions. However;among the studied stations the most distortions and most wind occurrence in winter belongs to Boroojen station with 56%, and Lordegan station with 76% wind calm has the calmest wind condition among other stations. Generally based on the mean of wind calm of the studied region, Boroojen station had 45% calm wind which shows the more wind occurrence in that station in comparison to other stations, and Lordegan station with yearly mean of 73% wind calm enjoys the calmest condition of wind.

In studying the annual mean temperature during this studying period, Koohrang station with mean of 9.8 ° C was identified as the coldest station and Lordegan with 17 ° C represented the warmest station of the region observed. But Koohrang station with mean of −2.3° C, Shahrekord with mean of 1.9° C, Boroojen with mean of 2 ° C, and finally Lordegan with mean of 6.7° C were respectively known as the coldest stations in winter.

Table 3 is set based on the new formula (formula No. 9). In this table, temperature is organized from +35 degree to -35 degrees of Fahrenheit at the first row, and wind speed is organized from 5m/h to 60m/h at the first column. Wind chill temperature is calculated from the related intersections of temperature and wind speed. This table is divided into areas that when human being is exposed to the conditions of each area, the condition becomes intolerable for him after a specific time period. For example, if wind chill temperature of one of the areas has dark color, the resistance threshold of this condition is only 5 minutes.

**Table 3:** wind chill temperature based on the formula No. 9 ^[14]^

Temperature (Fahrenheit)																
(Wind speed = m/h)
		**35**	**30**	**25**	**20**	**15**	**10**	**5**	**0**	**−5**	**−10**	**−15**	**−20**	**−25**	**−30**	**−35**
	**5**	31	25	19	13	7	1	−5	−11	−16	−22	−28	−34	−40	−46	−52
	**10**	27	21	15	9	3	−4	−10	−16	−22	−28	−35	−41	−47	−53	−59
	**15**	25	19	13	6	0	−7	−13	−19	−26	−32	−39	−45	−51	−58	−64
	**20**	24	17	11	4	−2	−9	−15	−22	−29	−35	−42	−48	−55	−61	−68
	**25**	23	16	9	3	−4	−11	−17	−24	−31	−37	−44	−51	−58	−64	−71
	**30**	22	15	8	1	−5	−12	−19	−26	−32	−39	−46	−53	−60	−67	−73
	**35**	21	14	7	0	−7	−14	−21	−27	−34	−41	−48	−55	−62	−69	−76
	**40**	20	13	6	−1	−8	−15	−22	−29	−36	−43	−50	−57	−64	−71	−78
	**45**	19	12	5	−2	−9	−16	−23	−30	−37	−44	−51	−58	−65	−72	−79
	**50**	19	12	4	−3	−10	−17	−24	−31	−38	−45	−52	−60	−67	−74	−81
	**55**	18	11	4	−3	−11	−18	−25	−32	−39	−46	−54	−61	−68	−75	−82
	**60**	17	10	3	−4	−11	−19	−26	−33	−40	−48	−55	−62	−69	−76	−84
	5 minutes		10 minutes		minutes											

According to Table 3, if wind chill temperature is considered in different areas, there will be some problems for human that are illustrated in Table 4, ^[7]^. After estimating WCI of winter by using formula 9 it became evident that except for Lordegan station in months of which no meaningful output of WCI was observed, in other stations a meaningful output of WCI was distinctively observed in January. Also in Koohrang station in February, meaningful outputs of WCI were observed. Only wind chill degrees of January are illustrated in Table 5. According to degrees derived from WCI, Koohrang station in January with mean of −28.75 was known as the coldest and roughest station and Lordegan station with mean of 71.69 was identified as the calmest station regarding WCI in Chaharmahal Bakhtiyari province.

**Table 4:** Wind chill temperature, problems and required actions^[12]^

Wind Chill Index	Cautionary Actions and Type of Comfort
**0 to −10**	Conditions for outdoor activities are discomforting and you should wear warm clothes.
**−10 to −15**	In this condition if body skin is not covered, it gets cold. You should wear warm wool clothes and gloves.
**−25 to −45**	All body organs should be covered. If some parts of body are not covered, it might get frosted and in long periods danger of sudden fall of body temperature is probable.
**−45 to −59**	This condition is very discomforting and outdoor activities should be limited to very short periods of time. There is danger of sudden fall of body temperature.
**−60 and above**	The outer condition is very dangerous. If body skin is not covered it will get frosted and the outdoor activity should be stopped and people should stay at home.

**Table 5:** Monthly index of wind chill in January for synoptic stations of Chaharmahal Bakhtiyari province

Year	Shahre Kord	Koohrang	Lordegan	Boroojen	Saman	Farokh Shahr	The average of total different stations
1990	−11.66	−35.15	-	−2.6	-	-	−16.5
1991	36.35	−10.92	-	22.8	-	-	16.1
1992	−31.52	−63.81	-	30.7	-	-	−21.5
1993	4.84	−49.09	-	42.5	-	-	−0.6
1994	41.71	1.64	-	−4.1	-	-	13.1
1995	54.62	−8.54	98.68	22.6	-	-	41.8
1996	−12.59	−49.27	50.54	14.2	-	-	0.7
1997	30.56	−34.71	78.07	27.6	-	-	25.4
1998	−8.13	−35.81	51.99	27.5	-	-	8.9
1999	39.59	−14.00	76.04	22.5	-	-	31.0
2000	33.43	−27.67	72.53	29.0	-	-	26.8
2001	26.11	−38.83	76.35	23.6			21.8
2002	34.57	−22.09	82.31	28.8			30.9
2003	18.91	−14.20	58.54	−3.8	32.51		18.4
2004	41.18	4.07	83.25	39.05	48.79		43.3
2005	−78.56	−43.14	60.24	−36.32	−51.79		−29.9
2006	−7.73	−39.57	50.41	−6.60	−7.97		−2.3
2007	−63.21	−59.09	43.12	−49.72	−55.73	−44.60	−38.2

The specified highlighted degrees indicate wind chill occurrence with high intensity degree and empty squares with inner white lines represent years without statistics of the stations investigated

## DISCUSSION

**Probability and return period of wind chill:** Analyzing meteorology data is not possible without conducting probable estimations. One of the technical words mostly used in statistics and probabilities is probability and return period of a specified parameter that occurs at a specified period of time. One of these significant meteorological phenomena is the estimation of return period and occurrence probability. Identifying return period and occurrence probability of wind chill is significant since by estimating its occurrence we could decrease its damages and effects in future specifically for athletes and people interested in winter sports who encounter cold climate more than others. This also could be used as a significant parameter for crisis management in order to protect athletes' health.

Therefore; for estimating wind chill occurrence probability in the considered periods from January 1990 to 2007, without considering the year of occurrence, wind chill data were organized in ascending order (from lowest to highest) and finally the occurrence probability of each wind chill degree was calculated through formula 10 named as Weibull:

Formula 10:

P=m/n+1

In this formula, **P** is occurrence probability, **m** is number of rows, and **n** is number of data^[15]^.

In order to summarize the final result in Table 6 the number of rows (m) and years were omitted. As a result in Table 6, following organizing wind chill degrees in an ascending order for each station, occurrence probability of different wind hill degrees were estimated. It should be mentioned that the output and occurrence probability of wind chills in Table 6 were not organized based on the order of years. In contrast, they were classified based on descending degrees of wind chills.

**Table 6:** Return period and occurrence probability and degrees of different wind chills for meteorology stations of Chaharmahal & Bakhtiyari

Shahrekord			Koohrang			Lordegan			Boroojen			Saman		
Ascendant amount of Wind chill	P	Return Period	Ascendant amount of Wind chill	P	Return Period	Ascendant amount of Wind chill	P	Return Period	Ascendant amount of Wind chill	P	Return Period	Ascendant amount of Wind chill	P	Return Period
−78.56	0.05	20	−63.81	0.05	20	-	-	-	−49.72	0.05	20	-	-	-
−63.21	0.11	9.09	−59.09	0.11	9.09	-	-	-	−36.32	0.11	9.09	-	-	-
−31.52	0.16	6.25	−49.27	0.16	6.25	-	-	-	−6.6	0.16	6.25	-	-	-
−12.59	0.21	4.76	−49.09	0.21	4.76	-	-	-	−4.1	0.21	4.76	-	-	-
−11.66	0.26	3.85	−43.14	0.26	3.85	-	-	-	−3.8	0.26	3.85	-	-	-
−8.13	0.32	3.13	−39.57	0.32	3.13	43.12	0.07	14.29	−2.6	0.32	3.13	-	-	-
−7.73	0.37	2.70	−38.83	0.37	2.70	50.41	0.14	7.14	14.2	0.37	2.70	-	-	-
4.84	0.42	2.38	−35.81	0.42	2.38	50.54	0.21	4.76	22.5	0.42	2.38	-	-	-
18.91	0.47	2.13	−35.15	0.47	2.13	51.99	0.29	3.45	22.6	0.47	2.13	-	-	-
26.11	0.53	1.89	−34.71	0.53	1.89	58.54	0.36	2.78	22.8	0.53	1.89	-	-	-
30.56	0.58	1.72	−27.67	0.58	1.72	60.24	0.43	2.33	23.6	0.58	1.72	-	-	-
33.43	0.63	1.59	−22.09	0.63	1.59	72.53	0.50	2	27.5	0.63	1.59	−55.73	0.13	8
34.57	0.68	1.47	−14.2	0.68	1.47	76.04	0.57	1.75	27.6	0.68	1.47	−55.73	0.25	4
36.35	0.74	1.35	−14	0.74	1.35	76.35	0.64	1.56	28.8	0.74	1.35	−51.79	0.38	2.67
39.59	0.79	1.27	−10.92	0.79	1.27	78.07	0.71	1.41	29	0.79	1.27	−51.79	0.50	2
41.18	0.84	1.19	−8.54	0.84	1.19	82.31	0.79	1.27	30.7	0.84	1.19	−7.97	0.63	1.60
41.71	0.89	1.12	1.64	0.89	1.12	83.25	0.86	1.16	39.05	0.89	1.12	32.51	0.75	1.33
54.62	0.95	1.05	4.07	0.95	1.05	98.68	0.93	1.08	42.5	0.95	1.05	48.79	0.88	1.14

Usually in meteorology the word Return Period is used very often. Return period is the opposite of probability and it is the number of years that is normally between two identical events. If return period is **T** and occurrence probability is **P**, then formula 11 is as follows:

Formula 11:

$$\text{T}=\frac{1}{\text{p}}$$

According to the formula 11, the amounts of return periods of different wind chills for stations of the studied region were estimated and the results are illustrated in Table 6. For instance, the return period of −49.72 wind chill of Boroojen station is every 20 years. It means that it is likely to have wind chills with intensity of −49.72 every 20 years or the return period of 54.62 wind chill output for Shahrekord station is estimated at 1.05 that indicates the occurrence of wind chill with this intensity for one time in a year. It means that every 105 years, the wind chill with this intensity occurs for 100 times.

## CONCLUSION

In this research the wind chill degrees of 6 stations from Chaharmahal Bakhtiyari province were estimated by using an innovative formula from NOAA Meteorology Service Center. Among the studied stations, Koohrang had the most intensive degrees of wind chill occurrence. The calmest condition was found in Lordegan. Therefore, athletes and tourists should use warmer clothes and covers in cold seasons in Koohrang in comparison with other studied regionsin order to protect themselves from the negative effects of sudden cold and occurrence of intense wind chills. However, in Lordegan station the wind chill occurrence is at the lowest level and maybe this region enjoys the highest comfort condition for body in regard to climatic features. Therefore, this region could be introduced as a suitable environment for athletic activities with the lowest negative effects of wind chill on athletes' health. A point to be considered is that the occurrence of wind chill was higher after the year 2000 compared to the period before this time. Therefore, if this process continues in the future, athletes should consider a higher probability of occurrence of more wind chills in the future in the considered regions. What is evident is the occurrence of intense wind chills with longer return periods in the studied region. However, the occurrence probability and the frequency of wind chill degrees with low intensity may show more reoccurrences in the future years.
